# Unusual lattice vibration characteristics in whiskers of the pseudo-one-dimensional titanium trisulfide TiS_3_

**DOI:** 10.1038/ncomms12952

**Published:** 2016-09-22

**Authors:** Kedi Wu, Engin Torun, Hasan Sahin, Bin Chen, Xi Fan, Anupum Pant, David Parsons Wright, Toshihiro Aoki, Francois M. Peeters, Emmanuel Soignard, Sefaattin Tongay

**Affiliations:** 1 School for Engineering of Matter, Transport and Energy, Arizona State University, Tempe, Arizona 85287, USA; 2 Department of Physics, University of Antwerp, Groenenborgerlaan 171, Antwerpen B-2020, Belgium; 3 Department of Photonics, Izmir Institute of Technology, Izmir 35430, Turkey; 4 LeRoy Eyring Center for Solid State Science, Arizona State University, Tempe, Arizona 85287, USA

## Abstract

Transition metal trichalcogenides form a class of layered materials with strong in-plane anisotropy. For example, titanium trisulfide (TiS_3_) whiskers are made out of weakly interacting TiS_3_ layers, where each layer is made of weakly interacting quasi-one-dimensional chains extending along the *b* axis. Here we establish the unusual vibrational properties of TiS_3_ both experimentally and theoretically. Unlike other two-dimensional systems, the Raman active peaks of TiS_3_ have only out-of-plane vibrational modes, and interestingly some of these vibrations involve unique rigid-chain vibrations and S–S molecular oscillations. High-pressure Raman studies further reveal that the A_g_^S–S^ S-S molecular mode has an unconventional negative pressure dependence, whereas other peaks stiffen as anticipated. Various vibrational modes are doubly degenerate at ambient pressure, but the degeneracy is lifted at high pressures. These results establish the unusual vibrational properties of TiS_3_ with strong in-plane anisotropy, and may have relevance to understanding of vibrational properties in other anisotropic two-dimensional material systems.

## Abstract (web summary)

Transition metal trichalcogenides are layered materials with strong in-plane anisotropy, and their vibrational characteristics remain largely unexplored. Here, the authors present high-pressure Raman studies to uncover unusual vibrational effects within this specific class of materials.

## Graphical abstract

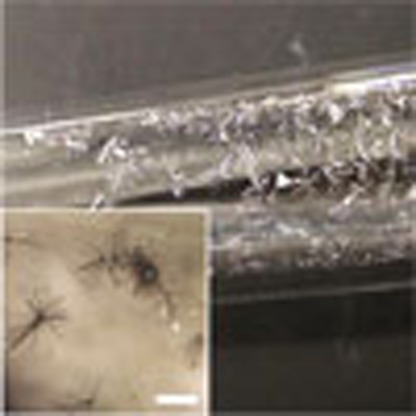

Two-dimensional (2D)-layered materials have emerged as a new class of materials with unusual optical, electrical, mechanical and thermal properties. Owing to their unique properties, they are of potential interest for applications for energy conversion, flexible electronics and information technologies^1^,^2^,^3^,^4^,^5^. Lamellar materials consist of two-dimensional (2D) layers weakly coupled through van der Waals (vdW) interaction; thus, they possess large structural anisotropy in the *c* axis (out-of-plane direction), whereas the individual 2D layer has high in-plane isotropy. However, there exist a small number of 2D materials in which one-dimensional (1D) chain-like dimerization takes place to induce strong in-plane anisotropy, such as few-layer black phosphorus^6^,^7^ and ReS_2_ (refs 8, 9). As a result of this anisotropy, their properties vastly differ in different crystalline (*a* versus *b* axis) directions, and offer unique functionalities compared with isotropic 2D materials^10^.

Layered group-IV transition metal trichalcogenides (TMTCs) with the chemical formula of MX_3_, for example, M=Zr, Ti and X=S, Se, are known to be anisotropic 2D systems^11^. Indeed, individual MX_3_ layers are made out of moderately interacting 1D-like chain structures, which result in large structural in-plane anisotropy^12^,^13^,^14^. Recently, titanium trisulfide (TiS_3_) attracted interest owing to theoretically calculated 1.0 eV direct gap values^15^,^16^,^17^,^18^, and field effect transistors have been demonstrated with relatively large electronic mobility^19^,^20^. Owing to its strong in-plane anisotropy, TiS_3_ nanosheets measure polarized carrier mobility of 80 cm^2^ V^−1^ s^−1^ along the *b* axis, whereas 40 cm^2 ^V^−1 ^s^−1^ along the *a* axis^21^. Despite these recent studies, its fundamental vibrational properties, that is, the origin and behaviour of experimentally measured Raman peaks and theoretically calculated vibrational dispersion, remain largely unknown. For example, all prominent TiS_3_ Raman modes are identified as A_g_-like ‘out-of-plane' vibrations without further insight into their origin and behaviour^22^,^23^. Lack of fundamental understanding of their vibrational properties limits our ability to explore their full potential in variety of applications and determination of their properties, such as thermal transport, interface interaction/physics, strain/mechanical behaviour and defect/impurity quantification.

In this work, we report on the unusual vibrational properties of TiS_3_ whiskers through pressure-dependent diamond anvil cell (DAC)-Raman spectroscopy measurements and density functional theory (DFT) calculations. In brief, results show that four prominent TiS_3_ Raman peaks located at ∼176, 298, 370 and 556 cm^−1^ are associated with rigid chain vibrations (I-A_g_^rigid^), internal out-of-plane vibrations constituting each monolayer (II-A_g_^internal^ and III-A_g_^internal^) and sulfur–sulfur diatomic motions (IV-A_g_^s–s^), respectively, which is in stark contrast to other isotropic 2D materials. Interestingly, DAC high-pressure Raman studies (up to ∼26 GPa) reveal that all Raman modes stiffen with pressure (d*ω/*d*P*>0) as commonly anticipated, except that the IV-A_g_^S–S^ mode softens (d*ω/*d*P*<0) because of weakening S–S interaction with pressure. Moreover, the II-A_g_^internal^ and IV-A_g_^s–s^ modes split into two individual peaks at pressures well below that required for inducing phase transition. Theoretical studies suggest that these two modes are degenerate at ambient pressure, but hydrostatic pressure lifts the degeneracy due to different d*ω*/d*P* values of each peak. This work, to our knowledge, marks the very first DAC measurements on TiS_3_ and introduces theoretically calculated phonon dispersion of TiS_3_ pseudo-1D systems. The findings also advance broader understanding of vibrational characteristics of TMTC materials with a potential impact on exploring and interpreting their thermal behaviour, mechanical properties, thermoelectric response and structural properties.

## Results

### TiS_3_ whisker growth and characterization

**Figure 1:**
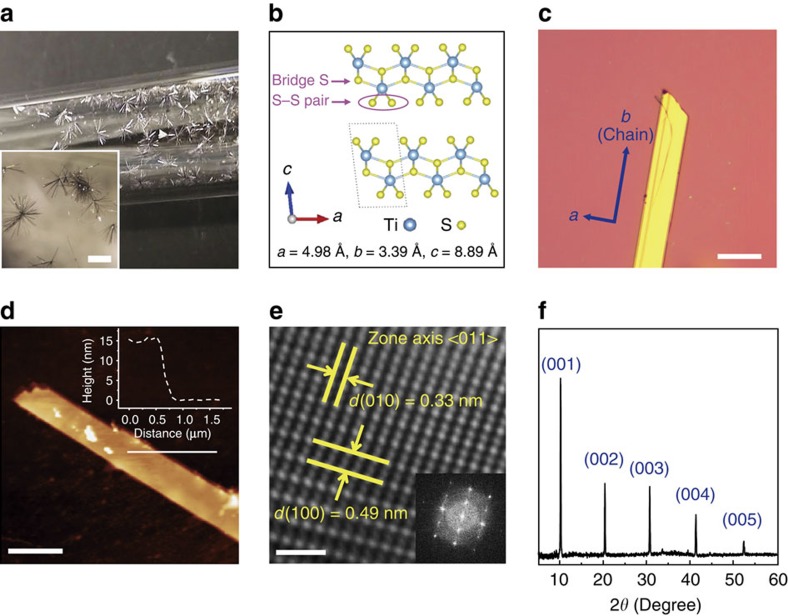
Material synthesis and characterization. (**a**) Optical photograph of as-prepared TiS_3_ whiskers in the sealed quartz ampule and (inset) zoom-in images of TiS_3_ whiskers that were grown at the interior surface of quartz. Scale bar, 250 μm. (**b**) Schematic cross-section schematic view of TiS_3_ chains along the *b* axis, with Ti atoms in blue and S atoms in yellow. (**c**) Optical image of typical TiS_3_ whiskers exfoliated on SiO_2_/Si substrates with large geometrical anisotropy along the *b* axis crystalline direction. Scale bar, 20 μm. (**d**) Atomic force microscope image of exfoliated TiS_3_ whiskers with thickness ∼15 nm. Scale bar, 1 μm. (**e**) High-resolution transmission electron microscopy image of TiS_3_ whiskers, and corresponding fast Fourier transform (FFT) image (inset). Scale bar, 1 nm. (**f**) X-ray powder diffraction pattern of TiS_3_.

TiS_3_ whiskers were grown directly from the interaction between titanium and sulfur in a sealed evacuated quartz ampule at 500 °C for 4 days via a chemical vapour transport process^13^,^24^. Typical growth yields TiS_3_ on the interior wall of the quartz ampule in a whisker form because of its highly anisotropic structure, and the whiskers measures from ∼200 to 500 μm in length (Fig. 1a) and only a few microns in width. In this work, most of the measurements were performed on flakes that measured ∼5 μm in width, >50 μm in length and ∼10–100 nm in thickness. TiS_3_ crystallizes in the monoclinic P2_1_/m space group with each unit cell containing two titanium atoms and six sulfur atoms by two distorted prisms, in which the titanium atom is at the top vertex of the prism, and the three sulfur atoms are at the three bottom vertices. The two prisms in the unit cell are also connected by binding the titanium atoms with their closest sulfur atoms in the neighbour prism (Fig. 1b). The lattice constants are calculated as *a*=4.98 Å, *b*=3.39 Å and *c*=8.89 Å. Here we note two types of sulfur atoms in a single prism: (1) the bridge S atom that is bonded to the two Ti atoms acting as a bridge and (2) the remaining two S atoms that are only bonded to only one Ti atom. To clarify, we differentiate these two types of sulfur atoms and label them as bridge sulfur and sulfur–sulfur pair when discussing the vibrational properties of TiS_3_. The repeating parallel chains of these triangular prisms are weakly coupled to each other via vdW interaction, giving rise to its signature structural in-plane anisotropy. Figure 1c,d shows the optical and atomic force microscopy (AFM) image of exemplary TiS_3_ whisker studied in this work. It is clear from these images that TiS_3_ whiskers have large geometric anisotropy, and the extending chains along the *b* axis are responsible for the anisotropic nature of the material. Figure 1e shows the high-resolution transmission electron microscopy (HR-TEM) image of the TiS_3_. The plane spacing measures ∼0.49 and ∼0.33 nm, which agrees well with the distance along the <100> and <010> lattice directions when the sample is aligned with the (011) crystal plane (JCPDS-ICDD 15-0783). The X-ray powder diffraction pattern (Fig. 1f) further confirms the high crystallinity of the synthesized TiS_3_.

### Vibrational properties and phonon dispersion

**Figure 2:**
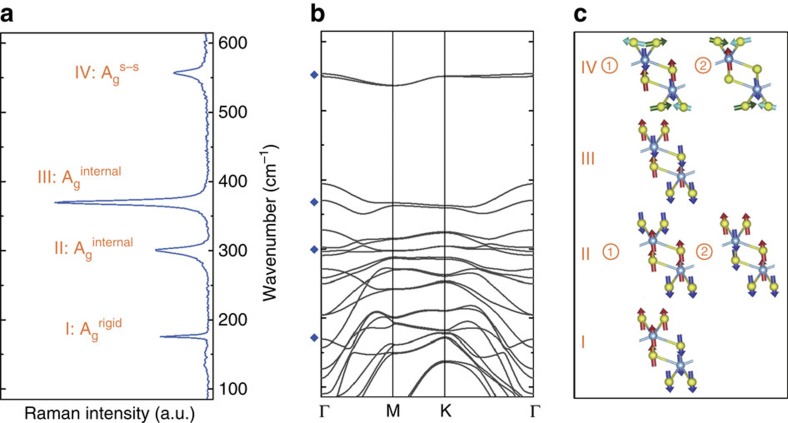
Vibrational properties and phonon dispersion of TiS_3_. (**a**) Raman spectrum of TiS_3_ whiskers measured in ambience at room temperature. (**b**) Phonon dispersion spectrum calculated using DFT of TiS_3_. The Raman active modes are highlighted by blue diamonds. (**c**) Corresponding Raman active optical modes in **a**.

Raman spectroscopy measurements reveal four prominent peaks located at 176, 298, 370 and 556 cm^−1^ (Fig. 2a). Since a primitive unit cell of TiS_3_ contains two titanium and six sulfur atoms, the phonon dispersion yields three acoustic and twenty-one optical modes in the first Brillouin zone as shown in Fig. 2b. These peaks match reasonably well with the calculated phonon dispersion spectrum, in particular to those Raman active branches of TiS_3_ whiskers highlighted by blue diamonds. To understand the vibrational nature of these Raman active modes, finite displacement method is performed and the characteristic atomic motion of each branch is shown in Fig. 2c.

Displacement method results show that lowest frequency peak (176 cm^−1^) originates from out-of-phase rigid vibration of each 1D-like TiS_3_ chain extending along the *b* axis (Fig. 2c labelled I). Since these chains move in the out-of-plane direction (*c* axis) and the atomic displacements within the chain is in-phase—keeping the rigidity of 1D chains owing to no relative displacement among TiS_3_ atoms—we label this peak as I-A_g_^rigid^. Overall, this mode can be pictured as two quasi-1D chains vibrating with respect to each other while keeping Ti–S bonding distance fixed within each chain. We note that this mode is absent in other 2D material systems such as transition metal dichalcogenides, TMDCs (MoS_2_, WSe_2_ and so on), post-transition metal chalcogenides (GaS and GaSe), and even highly anisotropic TMDCs (ReS_2_ and ReSe_2_) layers where Re atoms form Re–Re dimer chains.

In contrast to the rigid chain I-A_g_^rigid^ mode, the peaks at 298 cm^−1^ (II-A_g_^internal^) and 370 cm^−1^ (III-A_g_^internal^) involve vibration within each TiS_3_ layer, and these two vibrational modes are labelled as A_g_^internal^ in relation to internal vibration in each layer. A closer look at Γ∼300 cm^−1^ shows that two different optical branches coincide with each other. This implies that the II-A_g_^internal^ mode consists of two degenerate modes as shown in Fig. 2c. Here the main difference between mode II-A_g_^internal^- (at lower frequency) and mode II-A_g_^internal^- (at higher frequency) lies in the relative vibration direction between the Ti atoms, bridge S atoms and S–S pairs across the two prisms, as is shown in Fig. 2c. We note that the presence of two near degenerate Raman peaks results in much larger full-width half-maximum (FWHM) value (12.1 cm^−1^) compared with I-A_g_^rigid^ (3.3 cm^−1^) and III-A_g_^internal^ (7.9 cm^−1^). The large FWHM value of II-A_g_^internal^ is attributed to degeneracy (presence of multiple) of modes at ∼298 cm^−1^. Similar to II-A_g_^internal^, the III-A_g_^internal^ mode also involves vibration of atoms, making up the individual layers except that three S atoms (both the bridge S and S–S pair) vibrate oppositely with the Ti atom in a single prism, whereas the other prism moves in a central symmetry way with it.

In contrast to all the above modes, highest frequency peak at 556 cm^−1^ also appears doubly degenerate (Fig. 2b) with relatively large FWHM value (12.3 cm^−1^) as shown in Fig. 2a. Surprisingly, these peaks are predominantly made of in-plane out of phase motion of S–S pair and in part (minor) the out-of-plane motion of Ti and the bridge S atoms as shown in Fig. 2c. Considering the signature S–S pair vibration, this peak is labelled as IV-A_g_^S–S^.

### Pressure-dependent vibrational studies

**Figure 3:**
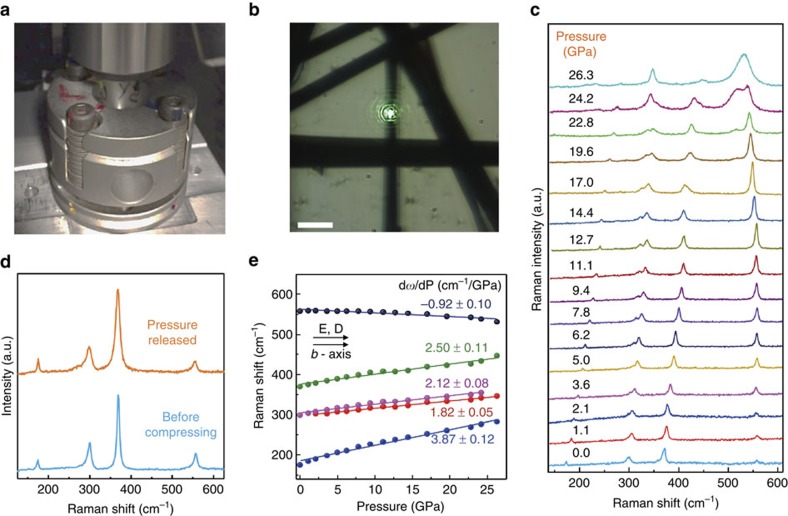
The vibrational properties of TiS_3_ under hydrostatic pressure. (**a**) Optical photograph of the DAC under × 50 optical lens in the experiment. (**b**) Optical image of TiS_3_ loaded inside the DAC. Scale bar, 1 nm. (**c**) Raman spectra of TiS_3_ under increasing hydrostatic pressure up to 26.3 GPa; plots are offset for clarity. (**d**) A comparison between the Raman spectra of TiS_3_ before applying pressure and after releasing pressure from 26.3 GPa. (**e**) Raman peak position as a function of pressure. The excitation (E) and detection (D) polarization is in parallel to the *b* axis. The dependence can be described as *ω* (*P*)=*ω*_*0*_+(d*ω*/d*P*)*P*. The *ω*_*0*_ and d*ω*/d*P* values are also shown in Table 1.

**Figure 4:**
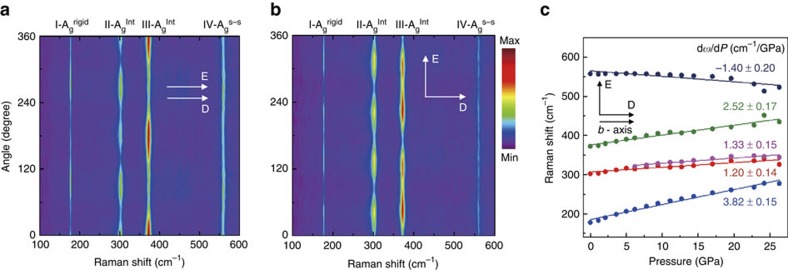
Polarization dependence of Raman modes and their pressure dependence. Angle-resolved Raman spectroscopy (2D contour plots) for all the Raman peaks in the (**a**) normal (E || D) and (**b**) orthogonal (E⊥D) configuration. E and D represents the excitation direction and detection direction, respectively. (**c**) Evolution of Raman spectrum in orthogonal configuration displaying similar pressure-dependent trends and d*ω/*d*P* values.

To further understand the vibrational properties, we have performed pressure-dependent Raman spectroscopy studies on TiS_3_ whiskers. Specifically, hydrostatic pressure was applied to the whiskers using a DAC, where the pressure media was a drop of methanol–ethanol (4:1) mixture and the pressure gauge was a small piece of ruby. Figure 3a displays the optical photograph of the DAC under the optical lens, and Fig. 3b shows the optical image of the TiS_3_ whiskers in the pressure media after loaded in the cell. Here the excitation (E) and detection (D) polarization was aligned parallel to the chain direction (E || D || *b* axis) to achieve high signal-to-noise ratio. Overall pressure trends, that is, negative d*ω*/d*P* values for IV-A_g_^S–S^, lifting the degeneracy of Raman peaks and overall pressure dependence, were found to be independent from the polarization direction, but for completeness orthogonal polarization (E ⊥ D || *b* axis) results are discussed and presented later in this section in Fig. 4c.

Figure 3c shows the pressure-dependent Raman spectra of TiS_3_ from ambient pressure up to 26.3 GPa in normal configuration (E || D || *b* axis). Rigid and internal modes, that is, I-A_g_^rigid^, II-A_g_^internal^ and III-A_g_^internal^, all stiffen with increasing pressure values because of increased interlayer interaction either stiffening the atomic bonds within each monolayers or increasing the interaction strength across 1D-like chain structures. The peak position of each individual mode scales linearly with pressure as depicted in Fig. 3e; thus, their frequency values can be described as *ω* (*P*)=*ω*_*0*_+(d*ω*/d*P*)*P*, where the slope value (d*ω*/d*P*) is given for each mode in Table 1. It is also noteworthy that the A_g_^Rigid^ mode is significantly more sensitive to applied pressure (d*ω*/d*P*(A_g_^rigid^)=3.82 cm^−1^/GPa>d*ω*/d*P*(A_g_^internal^)≈2 cm^−1^ GPa^−1^) compared with internal modes likely because of easiness to increase interaction between moderately interacting 1D-like chains—as opposed to strongly interacting atoms making up individual layers and chains. After the pressure is released, Raman spectrum of TiS_3_ returns back to its original (not pressurized) state, suggesting that pressure effects are reversible up to 26.3 GPa, which is the highest pressure that can be attained in our set-up.

In contrast to other Raman peaks, the IV-A_g_^S–S^ mode softens with pressure, that is, d*ω*/d*P*(A_g_^S–S^)<0, which is rather unique compared with observations made on other layered systems. What is the origin of negative d*ω*/d*P* value of A_g_^S–S^ mode? Since IV-A_g_^S–S^ is the only mode with finite in-plane vibrational contribution from S–S vibrations, it is possible that reduced interlayer distance at high pressures increases the S–S distance through an increase in S-Ti-S angle due to increase orbital interaction between adjacent layers. On the basis of this, we argue that the negative d*ω*/d*P* value is potentially related to softening of S–S vibration, reducing the vibrational frequency of the A_g_^S–S^ mode.

To further understand the mode stiffness, we also performed quantitate analysis by calculating the Grüneisen parameters^25^ of a vibration mode, *γ,* which is defines as *γ*=−(∂ln*ω*/∂ln*V*)=(*ωχ*_*T*_)^−1^(∂*ω*/∂*P*), where *ω* is the frequency of the specific mode of interest, *V* is the volume, *P* is the pressure and *χ*_*T*_=−*V*^−1^ (∂*V*/∂*P)* is the isothermal compressibility. In our case, the isothermal compressibility *χ*_*T*_ is replaced as −*c*^−1^(∂*c*/∂*P*) since our interested modes are along the *c* axis. Thus, the final expression of the Grüneisen parameter is expressed as *γ*=(−*ωc*^−1^(∂*c*/∂*P*))^−1^ (∂*ω*/∂*P*). The calculated Grüneisen parameters are also shown in Table 1. We conclude that the I-A_g_^rigid^ mode is much stiffer than other modes because of its rigid chain nature.

Here we note that, related to particular Raman tensor of this material system, Raman modes show large angular variation with polarization angle. This is apparent from angle-resolved Raman spectroscopy data presented in 2D contour plots in Fig. 4a,b in the normal (E || D) and orthogonal (E⊥D) configurations, respectively. The polarization angle is defined as the angle between the detection direction and *b* axis. Depending on the polarization angle and polarization configuration, Raman intensity of different peaks goes through maxima and minima, and these two- or fourfold symmetry (two- or four-lobed) features are associated with the particular Raman tensors specific to this material system. These findings are in line with a recent study^26^ on TiS_3_, which has shown similar polarization effects on the III-A_g_^Internal^ peak. In the same work, however, the other peaks demonstrated slightly less polarization-dependent response, in contrast to relatively strong polarization response measured in Fig. 4. This effect could possibly be attributed to presence of defects in the referenced work resulting in partial loss of crystalline anisotropy and much reduced angular response.

It is noteworthy to highlight that the polarization angle has no effect on the Raman peak position but has an impact on their relative intensity to each other as shown in Fig. 4a,b. Since internal mode (II- and III-A_g_^Internal^) peaks are harder to identify at high pressures, normal polarization configuration was selected in Fig. 3c,d to improve the signal from these modes (see Fig. 3c at 0 GPa and Fig. 4a). However, similar measurements in the orthogonal polarization direction also yield similar trends (that is, negative d*ω/*d*P* for IV-A_g_^s–s^ and peak splitting related to lifting degeneracy) and nearly matching d*ω/*d*P* values as shown in Fig. 4c. Similarly, the pressure dependence trends remain the same for thinner (∼ tens of nanometre thick) flakes as shown in Supplementary Fig. 1. However, we find it harder to collect sufficient and reliable signal at high pressures possibly because of the much weaker Raman intensity related to reduced material quantity as well as folding/wrinkling of ultrathin flakes in DAC liquid media causing unintentional flake folding.

### Lifting the degeneracy of Raman modes

**Figure 5:**
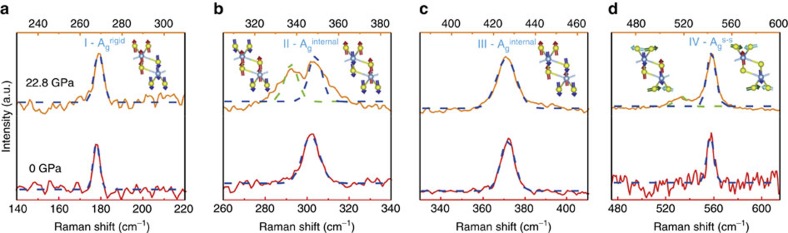
A comparison between the shape of each Raman peak at 0 and 22.8 GPa. (**a**) I-A_g_^rigid^ mode, (**b**) II-A_g_^internal^ mode, (**c**) III-A_g_^internal^ mode (**d**) and IV-A_g_^s–s^ mode.

In addition to the unusual pressure dependence of the A_g_^S–S^ mode, we note that some of the peaks split into two components going from ambient to high pressures. In Fig. 5, we compare the shape of each Raman peak at 0 and 22.8 GPa. This figure convincingly shows that only II-A_g_^internal^ and IV-A_g_^S–S^ modes split into two parts, whereas the other two retain their overall peak shape except minuscule broadening in their FWHM.

What is the origin of observed peak splitting for some of the peaks? This cannot be because of phase transition: peak splitting may (erroneously) be interpreted as ‘pressure-induced phase transition'. However, careful pressure-dependent DFT calculations show that material retains its symmetry up to 30 GPa (possibly above) and does not undergo phase transition. We, however, note that the pressure-induced splitting only occurs for doubly degenerate II-A_g_^internal^ and IV-A_g_^S–S^ modes (Fig. 2b). Since each mode, including the degenerate ones, has different frequency dependence with applied pressure (different d*ω*/d*P* values), we argue that mode-specific splitting effect can be attributed to lifting the degeneracy of II-A_g_^internal^ and IV-A_g_^S–S^ modes by applied pressure. More explicitly, initially degenerate modes are in close proximity to each other within ∼5 cm^−1^, causing II-A_g_^internal^ and IV-A_g_^S–S^ modes to have larger FWHM values compared with other modes; however, as the pressure is applied these modes start to separate from each other because of different d*ω*/d*P* values. Figure 5b further shows the splitting of the II-A_g_^internal^ mode; the FWHM values of the two peaks at 22.8 GPa, with the green dash line representing the II-A_g_^internal^- mode and the blue dash line representing the II-A_g_^internal^- mode, are comparable to that of the original degenerated II-A_g_^internal^ mode. Similarly, blue and green dash lines in Fig. 5d can be attributed to the degenerate modes forming the A_g_^S–S^ peak.

## Discussion

To the best of our knowledge, results mark the first high-pressure DAC, extensive Raman and theoretical investigation on pseudo-1D TiS_3_ material. Results are unexpected a *priori* as atomic motions of TiS_3_ vibrational modes are drastically different from other isotropic (MoS_2_, WSe_2_, and so on) and even anisotropic materials (ReS_2_, ReSe_2_ and black phosphorus). TiS_3_ exhibits various Raman active modes that are related to unique rigid chain vibrations (where chains vibrate as a whole), S–S molecular pair oscillations and conventional out-of-plane mode. Pressure-dependent DAC Raman studies reveal that (i) the A_g_^S–S^ mode has a unique response to applied hydrostatic pressure with negative d*ω*/d*P*<0 scaling and (ii) some modes appear to be degenerate, which is lifted only after pressure is exerted. Our theoretical studies by DFT and finite displacement methods further fundamental understanding of TiS_3_ and TMTC systems. Previously unknown vibrational properties of the material TiS_3_ may have broader impact impact towards understanding and interpreting thermal (thermal transport), structural (defects and impurities) and mechanical properties (strain) relevant for potential applications in optoelectronic and photonic devices.

## Methods

### Growth and characterization of TiS_3_ whiskers

TiS_3_ whiskers were grown through the direct reaction of titanium wire and sulfur following the stoichiometric ratio. One gram of precursors was placed inside an evacuated and sealed quartz tube at a vacuum level of ∼10^−5^ Torr. The inner diameter for the tube was 15 mm and the length of it was 210 mm. The loaded ampule was placed in a three-zone furnace and heated for 3 days. The end of ampule with source materials was heated up to 500 °C while keeping a temperature gradient of 100 °C at the other end. TiS_3_ whiskers were cleaved at the wall of the ampule at the cold end. The HR-TEM image was taken using JEOL JEM 2010 F at a voltage of 100 kV. The high crystallinity was further proved by the powder X-ray diffraction technique using Cu Kα irradiation on Siemens D5000 X-Ray diffractometer. The Raman spectroscopy measurements of TiS_3_ whiskers were taken using a Renishaw InVia Raman microscope under × 50 objective lens (numerical aperture=0.95) using 488 nm laser as the excitation source, and the laser power was set to 0.8 mW.

### High-pressure Raman spectroscopy

The hydrostatic pressure was applied using a DAC. The TiS_3_ sample was placed in the pinhole of a gasket with ∼200 μm diameter, where a drop of methanol–ethanol (4:1) mixture was used for pressure media. The pressure gauge was a small piece of ruby crystal deposited in the pinhole. Pressure was applied slowly to 26.3 GPa. The Raman spectra with increasing pressure were measured under long working distance × 50 optical lens (numerical aperture=0.42) using green laser (*λ*=532 nm) as the radiation source with a power of 1.3 mW. The data were collected using an Acton 300i spectrograph and a back thinned Princeton Instruments liquid nitrogen-cooled charge-coupled device detector.

### DFT calculations

The first-principles calculations were carried out in the framework of DFT as implemented in the Vienna *Ab initio* Simulation Package (VASP)^27^,^28^. Generalized gradient approximation (GGA) of Perdew–Burke–Ernzerhof was employed for the exchange and correlation potentials^29^. A 10 × 15 × 5 Γ-centred **k**-point mesh was used for the Brillouin zone integration for the primitive unit cell of bulk TiS_3_. Frozen-core projector-augmented wave^30^,^31^ potentials were used with 500 eV kinetic energy cutoff for the plane-wave expansion. The convergence criterion for energy is set to 10^−5^ eV between two consecutive steps in the self-consistent field calculations. The atomic positions were relaxed until the Hellmann-Feynman forces are less than 10^−4^ eV Å^−1^. We used the DFT-D2 method of Grimme as implemented in VASP in order to describe the vdW interaction between TiS_3_ layers correctly^32^. The vibrational spectrum of the systems was investigated via *ab initio* phonon calculations. These were performed in the harmonic approximation by making use of the small displacement method implemented in the PHON code^33^.

### Data availability

The data that support the findings of this study are available from the corresponding author on request.

## Additional information

**How to cite this article:** Wu, K. *et al*. Unusual lattice vibration characteristics in whiskers of the pseudo-one-dimensional titanium trisulfide TiS_3_. *Nat. Commun.* **7,** 12952 doi: 10.1038/ncomms12952 (2016).

## Supplementary Material

Supplementary Information Supplementary Figure 1

## Figures and Tables

**Table 1:** Quantitative analysis of vibration properties of TiS_3_.

**Vibration mode**	***ω***_**ambient**_ **(cm**^−**1**^**)**	**d*****ω*****/d*****P*** **(cm**^−**1**^** GPa**^**−1**^**)**	**Grüneisen parameter**
I-A_g_^rigid^	176	3.87±0.12	1.10±0.03
II-A_g_^internal^-	298 (Degenerate)	1.82±0.05	0.31±0.01
II-A_g_^internal^-		2.12±0.08	0.36±0.01
III-A_g_^internal^	370	2.50±0.11	0.34±0.01
IV-A_g_^s–s^-	556 (Degenerate)	—	
IV-A_g_^s–s^-		−0.92±0.10	−0.08±0.01

The assignments, ambient Raman peak position (*ω*_ambient_) and its pressure dependence (d*ω*/d*P*), and Grüneisen parameters of each vibration mode for TiS_3_ whiskers.
