# Supplementary material for: Unusual lattice vibration characteristics in whiskers of the pseudo-one-dimensional titanium trisulfide TiS3
Source: Nat Commun. 2016 Sep 22;7:12952. doi: 10.1038/ncomms12952 (PMC5036143; doi:10.1038/ncomms12952)
Supplement: Supplementary Information — Supplementary Figure 1 [file ncomms12952-s1.pdf]

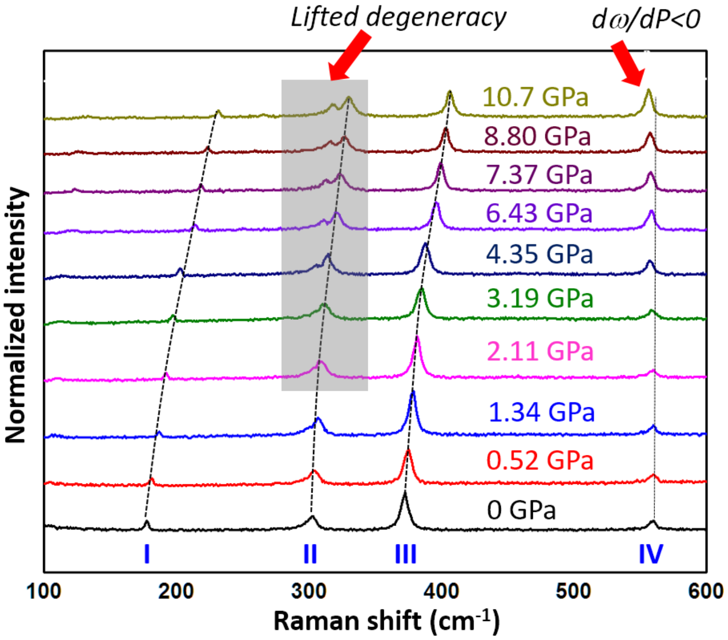

Supplementary Figure 1. DAC measurements on ultra-thin TiS_3 flakes. In the main manuscript, we have mentioned that similar degeneracy and negative dω/dP observation also apply to thin flake. In this figure, we show changes in the Raman spectrum of ultra-thin TiS_3 flakes (~10 nm in thickness) from ambient (0 GPa) to high (10.7 GPa) pressures. In summary, peak II's degeneracy splits in the 4-10 GPa range, peak IV has negative dω/dP dependence in line with similar measurements on thicker (~10s of nm in thickness) flakes. Here, we note that we observe peak splitting for peak III and peak IV on thicker flakes (see Figure 3c) above 17 GPa and 23 GPa respectively. Based on our measurements, it is rather difficult to attain such high pressures when ultra-thin flakes are in liquid media (inside DAC) due to folding, buckling, and twisting actions. Data taken below 10 GPa still shows trends similar to those observed in thicker samples. For example, peak II splits around 2 to 3 GPa range which exactly matches to Figure 3c. Similarly, peak IV also shifts to the lower frequencies with increasing pressure in line with observation made on thicker flakes (Figure 3c).
